# The Meridian Tropism and Classification of Red Yeast Rice Investigated by Monitoring Dermal Electrical Potential

**DOI:** 10.1155/2021/1696575

**Published:** 2021-08-20

**Authors:** Meng-Tian Wang, Qiao-Juan He, Jing-Ke Guo, Shu-Tao Liu, Li Ni, Ping-Fan Rao, Tian-Bao Chen, Sheng-Bin Wu, Shuai-Jun Zhao, Jia-Hui Qiao, Peng-Wei Zhang, Yu-Bo Li

**Affiliations:** ^1^Institute of Biotechnology, Fuzhou University, Fuzhou 350002, Fujian, China; ^2^School of Food Science and Biotechnology, Zhejiang Gongshang University, Hangzhou 310035, Zhejiang, China; ^3^Department of Food and Biological Engineering, Zhicheng College, Fuzhou University, Fuzhou 350002, Fujian, China; ^4^Institute of Food Science and Technology, Fuzhou University, Fuzhou 350108, Fujian, China; ^5^Natural Drug Discovery Group, School of Pharmacy, Queen's University Belfast, Belfast BT9 7BL, Northern Ireland, UK; ^6^Center for Preventive Treatment and Health Management, The Affiliated Hospital of Hangzhou Normal University, Hangzhou 310015, Zhejiang, China; ^7^College of Information Science and Electronic Engineering, Zhejiang University, Hangzhou 310027, Zhejiang, China

## Abstract

Red yeast rice is a traditional Chinese medicine and food that has been purported to color food, ferment, and lower cholesterol. In order to study the antioxidative capacity of red yeast rice and the effects on electrical potential difference (EPD) of 12 acupuncture meridians, the pH value, oxidation reduction potential (ORP), ABTS, FRAP, T-SOD, and particle size distribution of red yeast rice were analyzed. 20 volunteers were recruited and randomly divided into two groups, the red yeast rice group (10 g red yeast rice and 40 g water) and control CK group (50 g water). The left 12 acupuncture meridians' EPD was real-time monitored. Samples were taken at the 10th minutes. The whole procedure continued for 70 minutes. It is shown that the pH value of the red yeast rice was 4.22, the ORP was 359.63 mV, the ABTS was 0.48 mmol Trolox, the FRAP was 0.08 mmol FeSO_4_, the T-SOD was 4.71 U, and the average particle size was 108 nm (7.1%) and 398.1 nm (92.9%). The results of 12 acupuncture meridians' EPD showed that the red yeast rice can significantly affect the EPD of stomach, heart, small intestine, and liver meridians.

## 1. Introduction

As traditional Chinese medicinal material with dual functions of medicine and food, red yeast rice has been widely applied in food coloring, fermentation, cholesterol reduction, and other aspects [1]. In addition, the red yeast rice contains many bioactive substances, including multiple ketone isoflavones, plant sterols, and unsaturated fatty acids and [2]. The effect of red yeast rice cholesterol mainly comes from the monacolin K composition. It has a same kind of structure of the compound with lovastatin and contains anti-inflammatory and antioxidant activity of the material, such as sterol, dimer acid, and tannin [3].

Acupuncture meridians can be used as a reference index to observe the physiological effects of acupoint intervention and functional substances that can be used as medication and food [4, 5]. In traditional Chinese theories, meridian tropism refers to the selective therapeutic effects of a medication and food on a certain part of the body. A medication and food may elicit evident or specific therapeutic action on the pathological changes in one or several acupuncture meridians. Meridian tropism theory is most important because it has guided clinical practice for thousands of years in Eastern Asia [6]. In recent years, the explanation of meridian tropism theory by modern scientific techniques would facilitate the understanding and application of traditional Chinese medicine. The urinary excretion rate, loss in weight of rats, and the electrolyte levels in the plasma were measured in order to study the meridian tropism for Tinglizi (*Semen Lepidii Apetali*), Yiyiren (*Semen Coicis*), and Cheqianzi (*Semen Plantaginis*) [7]. The relationship between the modified Wuzi Yanzong prescription and meridian tropism was studied according to the tissue distributions of the metabolites, mainly flavanoid compounds in rats [8]. The distribution of tryptanthrin in rat tissues, following oral administration at a dose of 100 mg/kg, was studied in order to characterize the relationship with meridian tropism of indigo naturalis [9]. The dermal acupuncture meridian electrical potential difference (EPD) also can be real-time monitored to observe and compare various medications' and foods' meridian tropism, for volunteers' acupoint antioxidant intervention [10, 11] and taking medicine and food functional materials, such as *Cordyceps militaris* [12], *Inonotus obliquus* (Fr.) Pilat [13], *Dendrobium officinale* Kimura et Migo [14], herbal drink [15], tea [16, 17], and so on.

Therefore, in order to study the antioxidative capacity of red yeast rice and the effects on EPD of 12 acupuncture meridians (the meridian tropism of red yeast rice), the pH value, oxidation reduction potential (ORP), ABTS, FRAP, T-SOD, and particle size distribution of red yeast rice were analyzed and the left 12 acupuncture meridians EPD were real-time monitored. This study mainly conducted physical and chemical analysis of red yeast rice in vitro and found that the EPD of 12 acupuncture meridians changed differently after volunteers took red yeast rice to provide a new idea for the application and development of red yeast rice.

## 2. Materials and Methods

### 2.1. Materials

Red yeast rice (Fujian screen red Bio Technology Co., Ltd., China); the total antioxidant capacity detection kit (ABTS method) (Shanghai beyotime Biotechnology Co., Ltd., China, Batch No. 092518190324); the total antioxidant capacity detection kit (FRAP method) (Shanghai beyotime Biotechnology Co., Ltd., China, Batch No. 1113118190524); and superoxide dismutase (T-SOD) assay kits (Nanjing Jiancheng Bioengineering Institute, China, Batch No. 20190511) were used.

A CS-700 Y crusher (Wuyi Haina Electric Appliance Co., Ltd., China); pH meter (American EUTECH company, US); ORP (Oxidation Reduction Potential) Analyzers (ORP electrode InLab Redox, Mettler Toledo Instrument Shanghai Co., Ltd., China); CF 15 RX II high-speed centrifuge (HITACHI, Japan); Zetasizer Nano laser particle size analyzer (Malvern Instrument Co., Ltd., UK); NH-4 digital display electronic constant temperature water bath (Jiangsu Guohua Electric Appliance Co., Ltd., China); KQ 5200E type ultrasonic cleaner (Kunshan Ultrasonic Instrument Co., Ltd., China); UV-1700 UV spectrophotometer (Tsujima Corporation, Japan); FlexStation 3 Microplate Reader (Molecular Devices, Inc., US); Ag/AgCl disposable electrocardiogram (ECG) electrode (Shanghai Junkang Medical Equipment Co., Ltd., China); and RM 6240C Multi-channel Physiological Signal Acquisition and Processing System (Chengdu Instrument Factory, China) were used.

### 2.2. Human Subjects

This study was performed with central enrollment and allocation by the SIBS.CAS-Zhejiang Gongshang University Joint Centre for Food and Nutrition Research (research center). 20 healthy volunteers who met the inclusion criteria were recruited and screened as experimental subjects from October 2018 to July 2019. All volunteers signed the informed consent voluntarily under the condition that they could fully accept the experimental scheme. This experiment was approved by the Ethics Committee of the SIBS.CAS-Zhejiang Gongshang University Joint Centre for Food and Nutrition Research (31500685-3).

- Inclusion criteria: (1) age: >18 years, regardless of gender; (2) voluntarily signing the informed consent; (3) not taking breakfast before the test; and (4) keeping regular diet and rest.
- Exclusion criteria: (1) having kidney, liver, and cardiovascular disease or other serious diseases; (2) having bad habits, such as smoking, drinking, and drug abuse; (3) long-term drug treatment; (4) taking breakfast before the test; and (5) pregnant women and those with hyperactivity of fire due to yin deficiency.

### 2.3. Randomization

A computer-generated random number was assigned to each participant. Volunteers were randomly divided into two groups: the red yeast rice group (10 g red yeast rice and 40 g water) and the control CK group (50 g water), with the ratio of 1 : 1. The investigator had no clinical involvement in the trial.

### 2.4. Treatment Protocol

After the recruitment of volunteers was completed, it was uniformly stipulated that they should complete the monitoring in the morning from 8 : 30 am to 10 : 30 am. The changes of EPD were observed before and after the volunteers took the sample. This was repeated three times, with each time interval of more than 7 days. The volunteers were conscious, placed in a supine position, and asked to breathe calmly. The acupoints were localized according to World Health Organization standard acupoint locations in the Western Pacific Region [18]. The Source-Sea (Yuan-He) acupoint pairs of the 12 meridians on the left were selected as the measurement points of the meridian EPD, with the Sea acupoints (proximal) as the positive pole and the Source acupoints (distal) as the negative pole, respectively (Table 1) [10]. The hair on the selected detection acupoints was trimmed. After previously disinfected with medical alcohol, the acupoints were connected to a digital potentiometer via Ag/AgCl disposable ECG electrodes and the EPD of 12 meridians of volunteers was monitored in real time. When monitoring to 10 minutes, the prepared red yeast rice or water should be taken, and it should be taken within 5 min. A total of 70 min was monitored before and after the administration.

### 2.5. Sample Preparation

Weighing about 18 g of red yeast rice and grinding it several times with a grinder, the red yeast rice powder was obtained. After 60 mesh sieves, 10 g of it was accurately weighed and dissolved in 40 g of distilled water. The mixture was ultrasonized for 20 min and extracted by using a water bath thermostatic agitator for 1 hour at 37°C and 700 r/min and centrifuged at 25°C for 5000 r/min for 15 min; the supernatant was taken as the sample to be tested and stored in a refrigerator at −20°C for later use.

### 2.6. Determination of pH Value

The sample should be calibrated with a standard solution before testing. After calibration, the electrode is completely passed over the sample to be tested. When the data are stable, the reading is recorded, and the measurement is repeated three times to take the average value.

### 2.7. Determination of REDOX Potential (ORP)

An appropriate amount of the sample was placed in a beaker for determination. The sample was immersed in the electrode, and the determination was repeated three times to take the average value and the determination time should not exceed 3 min.

### 2.8. Antioxidant Activity Determination

The total antioxidant capacity (ABTS and FRAP) and SOD activity of samples were analyzed with colorimetric assay kits (Shanghai beyotime Biotechnology Co., Ltd., China, and Nanjing Jiancheng Bioengineering Institute, China).

### 2.9. Particle Size Distribution Determination

Red yeast rice solution was detected using a Zetasizer Nano particle size tester. 1 mL sample was taken and placed in the detection dish. The relevant parameters of the instrument were set as temperature 25°C, scattering angle 173°, and preheating time 2 min, and each cycle was scanned 3 times.

### 2.10. Statistical Analysis

The study design called for at least 20 healthy subjects. Sample size estimations were not performed. Because of the complex design of this pilot study, sample size was chosen on the basis of practical considerations. Therefore, this study was not designed to have sufficient power, and the results of statistical testing have to be interpreted as descriptive, explorative, and hypothesis generating rather than as confirmatory. Data are reported as means ± standard deviation. The statistical analysis was performed with IBM SPSS Statistics 22. Results in patients and healthy subjects were compared using the two-sample t-test.

## 3. Results

### 3.1. Influence of Red Yeast Rice on Meridian EPD

**Figure 1:**
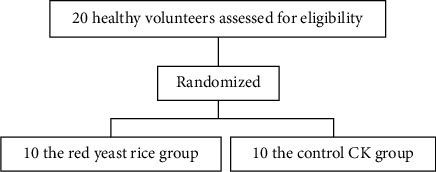
Study flow chart.

We screened 20 healthy volunteers into the study. All of them completed the study. The study flow is presented in Figure 1.

**Figure 2:**
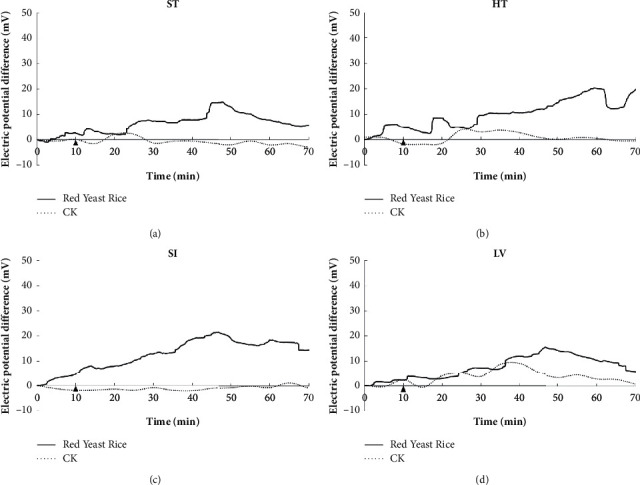
Electric potential difference of the stomach meridian (a), heart meridian (b), small intestine meridian (c), and liver meridian (d).

**Figure 3:**
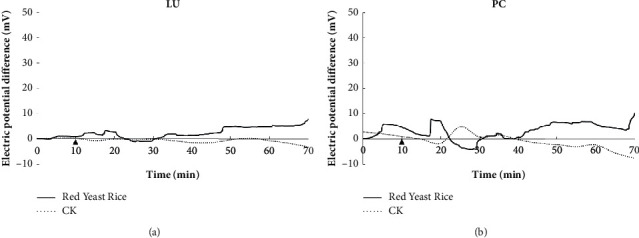
Electric potential difference of the lung meridian (a) and pericardium meridian (b).

**Figure 4:**
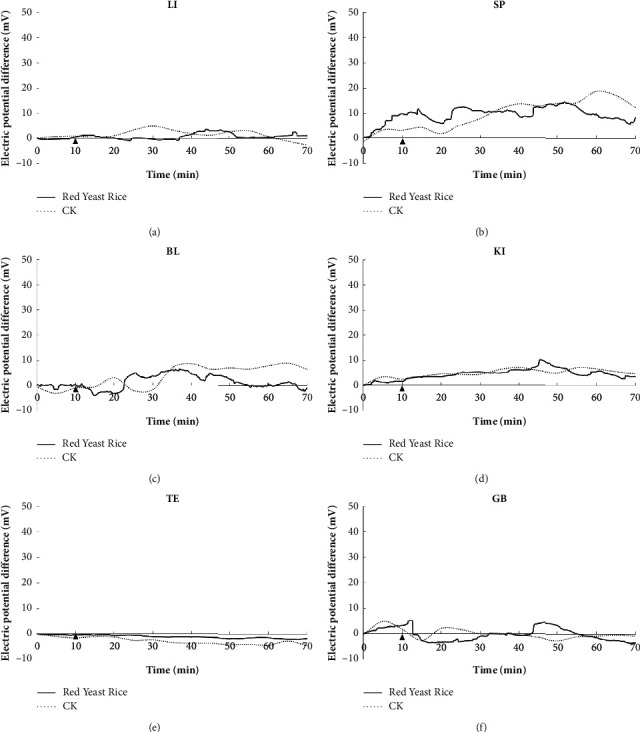
Electric potential difference of the large intestine meridian (a), spleen meridian (b), bladder meridian (c), kidney meridian (d), triple energizer meridian (e), and gallbladder meridian (f).

In this study, after volunteers took red yeast rice or water, the EPD of 12 meridians on the left side of volunteers was monitored in real time for 70 min. The results are shown in Figures 2–4. The EPD changes of the control CK group are relatively gentle, and the fluctuation range is within 10 mV. Red yeast rice had obvious effects on the EPD of stomach, heart, small intestine, and liver meridians. After the administration of red yeast rice, the fluctuation range of the meridian EPD of the volunteers exceeded 10 mV, the peaks of the stomach, small intestine, and liver meridians all appeared at 40–50 min, and the peaks of the heart meridians appeared at 50–70 min, as shown in Figure 1. For the lung meridian and pericardium meridian, the fluctuation range of the meridian EPD of the volunteers was about 10 mV (Figure 2).

The EPD fluctuation of the meridians of the large intestine, spleen, bladder, kidney, triple energizer, and gallbladder of volunteers in the red yeast rice group is not different from that in the control CK group, as shown in Figure 3.

### 3.2. Antioxidant Activity of Red Yeast Rice and Distilled Water

The pH value, REDOX potential value (ORP), ABTS, FRAP, and T-SOD of red yeast rice and distilled water were determined as shown in Table 2. Distilled water, as a solvent, has a certain REDOX potential value [19]. When the REDOX potential value of red yeast rice is lower than that of distilled water, it indicates that red yeast rice presents reductivity.

### 3.3. Particle Size Distribution of Red Yeast Rice

The average particle size of red yeast rice soup was 108 nm (7.1%) and 398.1 nm (92.9%), respectively.

## 4. Discussion

In our studies of reactive oxygen species (ROS) distribution in living animals by ROS indicator visualization, a few vertical fluorescent lines were demonstrated on the body. They could be almost perfectly superimposed on a standard human acupuncture meridian network [20, 21]. ROS has a strong connection with REDOX potential (ORP) and antioxidant activity. Moreover, topical application of antioxidants to acupoints was found to result in an acupuncture-like action [10, 11, 22, 23]. All those results point to a new applaudable hypothesis of meridians as channels of ROS and ROS modulation along meridians as the acupuncture mechanism.

Although in Table 2, the results of antioxidant activity of red yeast rice in vitro show weak acidity (pH = 4.22 ± 0.19, high H^+^ solubility, and partial oxidation), its overall REDOX potential was lower than that of distilled water and showed high reductivity. The results of ABTS, FRAP, and T-SOD all showed that red yeast rice had antioxidant activity. The particle size distribution of red yeast rice soup was determined. The particle size distribution was mainly divided into two components. The main component was 398.1 nm (92.9%), followed by 108 nm (7.1%). The active ingredients isolated from *Semen Armeniacae Amarum* with particle size less than 200 nm had strong biological activity [24]. In addition, enzymatically active nanoparticles of Cu/Zn SOD were simultaneously generated during the reaction, with an average particle size of 175.86 ± 0.71 nm [25]. The nanoparticles isolated from sun-dried *Isatis indigotica* Fort. root decoction were also about 120 nm [26]. The nanoparticles less than 200 nm can enter the macrophages, significantly reduce the oxidative stress level of cells, and show the strong oxidation resistance [27], with the effect of detoxification [28].

When substances with antioxidant activity are introduced into the human body, they will cause changes in the content of some free radicals to give full play to their antioxidant effect, thus causing changes in the meridian EPD value [10–17]. Compared with other acupoints, the EPD signal-to-noise ratio between the 12 meridians on the left side of the Source-Sea (Yuan-He) acupoint is higher and suitable for monitoring [10]. The administration of red yeast rice can cause significant changes in the meridians of the stomach, heart, small intestine, and liver, mild and moderate in the lung meridian and pericardium meridian, but has no obvious effect on the others. Medical scientists in the past dynasties summarized the natural taste and meridian tropism of red yeast rice, concluding that it has sweet taste, smooth nature, and nontoxic. The meridian tropism of red yeast rice is the meridian of the large intestine, spleen, and liver [29]. Modern pharmacology has shown that red yeast rice exerts potential protective effects on the liver, pancreas, blood vessels, and intestines [2], which matches the functions to the meridians of the liver, stomach, heart, and small intestine, respectively. Therefore, the results of this study have reference significance for the treatment of diseases related to stomach, heart, and small intestine meridians by red yeast rice beyond the meridian of the large intestine, spleen, and liver [29].

The meridian orientation of traditional Chinese medicine refers to the selective effect of traditional Chinese medicine on different organs and the specificity of traditional Chinese medicine on meridians based on the theory of meridians [6–9]. The meridian tropism has been proved to be effective in guiding clinical practice, but other than the scientifically mysterious bioenergy or *Qi* concept, there is hardly any clue to what exactly happens immediately after a medication and could be observed precisely. It is still difficult to determine how red yeast rice selectively influences the electrical properties of particular meridians; however, this study attempts to explore the physiological effects of red yeast rice on the human body by monitoring the meridian EPD, so as to provide new ideas for the analysis of the specificity of red yeast rice on the meridian, the application, and development of red yeast rice.

## 5. Conclusions

In this study, the pH value of the red yeast rice was 4.22, the ORP was 359.63 mV, the ABTS was 0.48 mmol Trolox, the FRAP was 0.08 mmol FeSO_4_, the T-SOD was 4.71 U, and the average particle size was 108 nm (7.1%) and 398.1 nm (92.9%). The results of 12 acupuncture meridians EPD showed that the red yeast rice can significantly affect the EPD of stomach, heart, small intestine, and liver meridians.

## Figures and Tables

**Table 1:** Acupuncture point selection of acupuncture meridians.

Meridian	Sea (He) acupoints	Source (Yuan) acupoints
Lung meridian	Chize LU5	Taiyuan LU9
Large intestine meridian	Quchi LI11	Hegu LI14
Stomach meridian	Zusanli ST36	Chongyang ST42
Spleen meridian	Yinlingquan SP9	Taibai SP3
Heart meridian	Shaohai HT3	Shenmen HT7
Small intestine meridian	Xiaohai SI8	Wangu SI4
Bladder meridian	Weizhong BL40	Jinggu BL64
Kidney meridian	Yingu KI10	Taixi KI3
Pericardium meridian	Quze PC3	Daling PC7
Triple energizer meridian	Tianjing TE10	Yangchi TE4
Gallbladder meridian	Yanglingquan GB34	Qiuxu GB40
Liver meridian	Ququan LR8	Taichong LR3

**Table 2:** Antioxidant activity of red yeast rice and distilled water.

Determination method	Red yeast rice	Distilled water
PH	4.22 ± 0.19^*∗∗*^	7.01 ± 0.01
Redox potential (mV)	359.63 ± 6.68^*∗∗*^	512.70 ± 9.36
ABTS (equal to the amount of Trolox standard solution substance) (mmol)	0.48 ± 0.11^*∗∗*^	0.02 ± 0.00
FRAP (amount of FeSO_4_ standard solution substance) (mmol)	0.080 ± 0.004^*∗∗*^	0.01 ± 0.00
T-SOD (U)	4.71 ± 0.19^*∗∗*^	0.09 ± 0.00

Note: compared with distilled water, ^*∗*^*P* < 0.05, ^*∗∗*^*P* < 0.01. *n* = 5.

## Data Availability

The data that support the findings of this study are openly available in Mendeley Data at https://dxdoi.org/10.17632/txc5g64t6s.1.
